# Flavanol Bioavailability in Two Cocoa Products with Different Phenolic Content. A Comparative Study in Humans

**DOI:** 10.3390/nu11071441

**Published:** 2019-06-26

**Authors:** Miren Gómez-Juaristi, Beatriz Sarria, Sara Martínez-López, Laura Bravo Clemente, Raquel Mateos

**Affiliations:** Department of Metabolism and Nutrition, Institute of Food Science, Technology and Nutrition (ICTAN-CSIC), Spanish National Research Council (CSIC), José Antonio Novais 10, 28040 Madrid, Spain

**Keywords:** flavanols, soluble cocoa products, bioavailability, human, plasma nutrikinetics, liquid chromatography coupled to electrospray ionisation and quadrupole time-of-flight mass spectrometry (LC-ESI-QToF-MS), colonic bacteria

## Abstract

Cocoa has beneficial health effects partly due to its high flavanol content. This study was aimed at assessing the absorption and metabolism of polyphenols in two soluble cocoa products: a conventional (CC) and a flavanol-rich product (CC-PP). A crossover, randomized, blind study was performed in 13 healthy men and women. On two different days, after an overnight fast, volunteers consumed one serving of CC (15 g) or CC-PP (25 g) in 200 mL of semi-skimmed milk containing 19.80 mg and 68.25 mg of flavanols, respectively. Blood and urine samples were taken, before and after CC and CC-PP consumption, and analyzed by high-performance liquid chromatography coupled to electrospray ionisation and quadrupole time-of-flight mass spectrometry (HPLC-ESI-QToF-MS). Up to 10 and 30 metabolites were identified in plasma and urine, respectively. Phase II derivatives of epicatechin were identified with kinetics compatible with small intestine absorption, although the most abundant groups of metabolites were phase II derivatives of phenyl-γ-valerolactone and phenylvaleric acid, formed at colonic level. 5-(4′-Hydroxyphenyl)-γ-valerolactone-sulfate could be a sensitive biomarker of cocoa flavanol intake. CC and CC-PP flavanols showed a dose-dependent absorption with a recovery of 35%. In conclusion, cocoa flavanols are moderately bioavailable and extensively metabolized, mainly by the colonic microbiota.

## 1. Introduction

Cocoa remains a popular foodstuff worldwide. Soluble cocoa products have been popular in Spain, among other countries, being commonly consumed twice a day, at breakfast and as part of an afternoon break known as “merienda”. The potential health-promoting effects of cocoa products have gained extensive attention in the last few years. Most of these effects are attributed to the polyphenolic fraction of cocoa, mainly flavanols epicatechin and catechin, and low molecular weight procyanidins such as procyanidins B1 and B2 [1,2]. In fact, cocoa has been defined as a functional food due to its high flavanol content [1]. Moreover, soluble cocoa products are also a source of methylxanthines (mainly theobromine), magnesium and dietary fiber; all are biologically active substances that may also affect human health positively [2].

The biological activity of phenolic compounds depends on their bioavailability and metabolic fate, as well as on their digestive accessibility, which is determined by the release from the food matrix and efficiency in trans-epithelial passage. Recently Mena et al. [3] reviewed the rate and extent of absorption of cocoa polyphenols in humans, as well as the metabolic pathways involved. These polyphenols are partially absorbed in the upper gastrointestinal tract, being conjugated by phase II enzymes into methoxy, sulfated and/or glucuronidated metabolites, with maximum plasma concentrations around 2 h after cocoa intake in the nM range [4]. Controversy exists regarding whether procyanidins can be broken down in the stomach yielding monomers that may be absorbed or, conversely, whether intact procyanidins can be absorbed from the gastrointestinal tract. In this sense, Ottaviani et al. [5] observed that dietary procyanidins do not contribute to the systemic pool of flavanols in humans when test drinks that contained only flavanols, flavanols and procyanidins, or only procyanidins were consumed by humans.

Polyphenols not absorbed in the small intestine reach the colon where they are metabolized by the intestinal microbiota mainly to phenyl-γ-valerolactone and phenylvaleric acid metabolites [3]. These ring fission products, as well as phenylvaleric acid conjugates, were excreted primarily 5–10 h after ingestion of green tea [6,7]. In a recent report on the absorption, distribution, metabolism, and excretion in humans of radiolabeled and stereochemically pure [2-^14^C](-)-epicatechin ([^14^C]epicatechin) [8], 20 different metabolites were identified and quantified: phase II derivatives of epicatechin, hydroxyphenyl-γ-valerolactones and phenylvaleric acids. Moreover, it was confirmed that the gut microbiome is a key driver of epicatechin metabolism. 

Nowadays, innovation in the elaboration of cocoa products includes using new delivery forms in an attempt to increase polyphenols’ bioavailability, like microencapsulation of cocoa phenols in cocoa-nut creams [9]. New soluble cocoa products enriched with bioactive components such as dietary fiber, methylxanthines or polyphenols are also being introduced into the food market. This is the case of two novel soluble cocoa products, produced and commercialized by the same manufacturer as the products used in the present study. One is rich in dietary fiber and the other rich in cocoa, containing 1.16 and 3.02 mg/g of flavanols, respectively [10]—amounts similar to the flavanol content of the soluble cocoas used in the present study (see below). When consumed in realistic doses by healthy and subjects at cardiovascular risk (hypercholesterolemic), results showed that both soluble cocoa products had a positive effect on serum lipid profile, increasing HDL-cholesterol without inducing anthropometric changes. These effects could be associated in part to the flavanol content in the two commercial cocoa products, although in that study the circulating levels of phenolic metabolites was not quantified. However, other studies have shown a correlation between the effect of dark chocolate (50 g, providing 7.5 gallic acid equivalents (GAE) of polyphenols) improving platelet function and the increased plasma concentrations of structurally-related (epi)catechin metabolites (SREM). These data confirm that the potential health benefits of cocoa consumption may be mediated by flavan-3-ol circulating metabolites [11], in spite of the limited bioavailability of cocoa polyphenols. 

Indeed, many studies have focused on determining the bioavailability and metabolism of cocoa phenolic compounds, although most bioavailability studies have been carried out with unrealistic doses (for some reviews see [3,12,13,14]). Therefore, the aim of the present work was to evaluate the bioavailability of flavanols in healthy humans after consuming a realistic amount of two soluble cocoa products: a conventional soluble cocoa (CC) and a flavanol-rich soluble cocoa (CC-PP). In addition, an important effort has been made to identify microbiota-derived metabolites, showing the importance of gut bacteria on polyphenol absorption and metabolism. 

## 2. Materials and Methods

### 2.1. Chemical Reagents and Materials

The commercialized soluble cocoa products used in the study were provided by Idilia Foods (previous company name: Nutrexpa S.L.), one being a conventional soluble cocoa product, labeled as CC, and the other a flavanol-rich soluble cocoa product, labeled as CC-PP. All solvents and reagents were of analytical grade unless otherwise stated. Ascorbic acid, epicatechin, catechin, procyanidin B1, 3-(3,4-dihydroxyphenyl)propionic acid, 3-(4-hydroxyphenyl)propionic acid, 3-(4-hydroxy-3-methoxyphenyl)propionic acid, 3,4-dihydroxyphenylacetic acid, 4-hydroxyphenylacetic acid, 4-hydroxy-3-methoxyphenylacetic acid, protocatechuic acid, 4-hydroxybenzoic acid and ferulic acid were from Sigma-Aldrich (Madrid, Spain). Procyanidin B2 and epicatechin-3-gallate were acquired from Extransynthese Genay Cedex (France). Methanol, formic acid, and acetonitrile (high-performance liquid chromatography (HPLC) grade) were acquired from Panreac (Madrid, Spain).

### 2.2. Quantification of Total Polyphenols of the Soluble Cocoa Products. Characterization and Quantification by high-performance liquid chromatography-mass spectrometry (HPLC-MS) and high-performance liquid chromatography-diode array (HPLC-DAD).

Total polyphenols were measured spectrophotometrically using the Folin–Ciocalteau reagent and gallic acid as standard. Results were expressed as µg equiv gallic acid/g product.

As described by Bravo et al. [15], cocoa extracts were obtained by washing 1 g of defatted cocoa with 40 mL of 50% aqueous methanol (HPLC grade) containing 0.8% of 2 mol/L hydrochloric acid for 1 h at room temperature with constant shaking. Afterwards, samples were centrifuged (10 min, 3000× *g*) and supernatants were collected. The residues obtained were successively extracted with 40 mL of 70% acetone (*v*/*v*) in water (1 h, constant shaking). The samples were then centrifuged (10 min, 3000× *g*) and supernatants were collected. Finally, supernatants obtained after each extraction step were combined and made up to 100 mL. Polyphenolic composition was analyzed using an Agilent 1200 series liquid chromatographic system equipped with an autosampler, quaternary pump, diode array detector (DAD) and simple quadrupole (sQ) mass spectrometer (Agilent Technologies, Waldbronn, Germany). Samples (20 μL) were injected into a Superspher RP18 column (4.6 mm × 250 mm i.d., 4 μm; Agilent Technologies) preceded by an ODS RP18 guard column kept in a thermostatic oven at 37 °C.

Elution was performed at a flow rate of 0.6 mL/min using a binary system consisting of 1% formic acid in deionized water (solvent A) and 1% formic acid in acetonitrile (solvent B). The solvent gradient changed from 6% to 10% solvent B over 20 min, 10% to 13% solvent B over 5 min, 13% to 15% solvent B over 5 min, 15% to 10% in 10 min, 10% to 6% in 5 min and then maintained isocratically for 5 min. Chromatograms were recorded at 280 nm. The mass spectrometer was fitted to an atmospheric pressure electrospray ionization (ESI) source, which operated in negative ion mode. Capillary voltage was set to 3500 V, with nebulizing gas flow rate of 12 h/L, drying gas temperature of 350 °C and nebulizer pressure of 45 psi. Mass spectrometry data were acquired in scan mode (mass range *m*/*z* 100–1000). Data acquisition and analysis were carried out in an Agilent ChemStation.

An Agilent 1200 series liquid chromatographic system (Agilent Technologies) equipped with a quaternary pump, column oven, autosampler and DAD was used to quantify the identified polyphenols in soluble cocoa products by high-performance liquid chromatography (HPLC–DAD). The chromatographic conditions (column, guard column, binary gradient, injection volume, etc.) were as described above. For quantitative analysis the external standard method was used. Samples were prepared and analyzed in triplicate and the results were expressed as the mean value.

### 2.3. Subjects and Study Design

The study protocol was conducted in accordance with the ethical recommendations of the Declaration of Helsinki and approved by the Ethics Committee of Hospital Universitario Puerta de Hierro in Majadahonda (Madrid, Spain) (ACT ID 256, 28th of June 2010; Project Identification Code AGL2015-69986-R). Recruitment of volunteers was carried out through placing advertisements at the Institute of Food Science, Technology and Nutrition (ICTAN). 

The study was carried out in thirteen healthy subjects (3 men and 10 women); the mens’ average age and body mass index were 26.67 ± 3.21 year and 22.47 ± 2.97 kg/m^2^, respectively, and womens’ were 32.60 ± 9.85 year and 23.36 ± 3.73 kg/m^2^, respectively. They were non-smoker, non-vegetarian, non-pregnant women, who were not taking any medication or nutritional supplements, and were not suffering from any chronic pathology or gastrointestinal disorder. The sample size was estimated attending to similar previous bioavailability studies [4,16]. The volunteers gave their informed consent prior to participation.

The present randomized, single-blind study was carried out at the Human Nutrition Unit (HNU) of the Institute of Food Science, Technology and Nutrition (ICTAN). Volunteers attended the HNU on two days, separated by two weeks. Three days previous to each visit, participants were instructed not to consume cocoa products (chocolate, soluble cocoa, etc.), juices, tea, wine, grape must, oranges, tangerines, apples, grapes, strawberries or other berries, beets, onions, soybeans and soy derivatives. In addition, volunteers were asked to complete a 24 h food intake recall the day before they attended the HNU in order to monitor any possible food restriction incompliance.

On each intervention day, volunteers arrived at the HNU after an overnight fast. Prior to the intake of the soluble cocoa product, a nurse inserted a cannula in the cubital vein of the non-prevailing arm of the volunteers and blood samples were collected into EDTA-coated tubes at baseline (*t* = 0) and 0.5, 1, 2, 3, 4, 6, and 8 h after consuming the corresponding cocoa product. The soluble cocoa products, either 15 g of CC or 25 g CC-PP, were dissolved in 200 mL of semi-skimmed milk following manufacturer’s preparation instructions. Plasma was separated by centrifugation (10 min, 3000 rpm, 4 °C) and stored at −80 °C until further analysis. Urine samples were collected at different time intervals (t = −2–0, 0–4, 4–8, 8–12, and 12–24 h) in urine collection flasks that contained 0.5 g of ascorbic acid as preservative and were aliquoted and frozen at −20 °C until analysis. A polyphenol-free breakfast, lunch and afternoon snack were provided 2 h, 4 h, and 8 h after consumption of cocoa products, and water and isotonic beverages were available *ad libitum*.

### 2.4. Extraction of Phenolic Metabolites from Biological Samples 

A liquid–liquid extraction and protein precipitation with acetonitrile was used to isolate metabolites from plasma. A 1 mL defrosted plasma sample was mixed with 50 μL of ascorbic acid (0.2 g/mL). After vortexing the aqueous mixture, it was added drop wise to 750 μL of cold acetonitrile and vortexed for 2 min before centrifuging at 12,000 rpm for 10 min at 4 °C. The supernatant was separated, and the pellet was re-extracted twice more following the same procedure. Supernatants were combined and reduced to dryness under a stream of nitrogen at 30 °C. The dried samples were resuspended in 150 μL of aqueous formic acid (0.1%) containing 10% acetonitrile acidified with 0.1% formic acid and centrifuged at 4 °C for 20 min at 14,000 rpm. The final supernatant was collected, filtered (0.45 μm pore-size, cellulose-acetate membrane filters, Albet, Dassel (Germany)) and 30 μL were analyzed by high-performance liquid chromatography coupled to electrospray ionisation and quadrupole time-of-flight mass spectrometry (HPLC-ESI-QToF-MS). Recoveries of the standards used to quantify metabolites ranged from 95 to 99%. 

Urine samples were diluted with an equivalent volume of Milli-Q water (50%) and centrifuged at 14000 rpm (10 min, 4 °C). Supernatants were filtered (0.45 μm pore-size cellulose-acetate membrane filters) and a 5 µL aliquot was directly injected into the LC-ESI-QToF-MS equipment.

### 2.5. Metabolite Identification by HPLC-ESI-QToF-MS Analysis

Analyses were performed on an Agilent 1200 series LC system coupled to an Agilent 6530A Accurate-Mass Quadrupole Time-Of-Flight (Q-ToF) with ESI-Jet Stream Technology (Agilent Technologies). Compounds were separated on a reverse-phase Ascentis Express C18 (15 cm × 3 mm, 2.7 m) column (Sigma-Aldrich Química, Madrid) preceded by a Supelco 55215-U guard column at 30 °C. The test samples, either 30 μL of the plasma extract or 5 μL of diluted urine, were injected and separated using a mobile phase consisting of Milli-Q water (phase A) and acetonitrile (phase B), both containing 0.1% formic acid, at a flow rate of 0.3 mL/min. The mobile phase was initially programmed with 90% of solvent A and 10% of B. The elution program increased to 30% of solvent B in 10 min. Then, the initial conditions (10% solvent B) were recovered in 5 min and maintained for 5 min. The Q-ToF acquisition conditions were as follows: drying gas flow (nitrogen, purity > 99.9%) and temperature were 10 L/min and 325 °C, respectively; sheath gas flow and temperature were 6 L/min and 250 °C, respectively; nebulizer pressure was 25 psi; cap voltage was 3500 V and nozzle voltage was 500 V. Mass range selected was from 100 up to 970 *m*/*z* in negative mode and fragmentor voltage of 150 V. Data were processed in a Mass Hunter Workstation Software. 

Due to the lack of standards for certain phase II metabolites, they were tentatively quantified using the calibration curves of their corresponding phenolic precursors. Thus, epicatechin was used to quantify epicatechin and 5-(3′,4′-dihydroxyphenyl)-γ-valerolactone (DHPVL) derivatives and 3,4-dihydroxyphenylpropionic acid to quantify phenylvaleric acid derivatives. The rest of microbial metabolites identified, derivatives of hydroxyphenylpropionic, hydroxyphenylacetic, hydroxybenzoic and hippuric acids, were quantified using their respective commercially available standards. Urine concentration of the excreted metabolites was normalized by the volume excreted in each studied interval. A linear response was obtained for all the standard curves (from 1 to 1,000 nM), as checked by linear regression analysis. Calibration curves were freshly prepared in a pool of both plasma and urine due to matrix effects. Limits of detection and quantification in plasma ranged from 1 to 5 nM and from 2 to 8 nM, respectively, while limits of detection and quantification in urine ranged from 2.5 to 30 nM and from 50 to 90 nM, respectively. The inter- and intra-day precision of the assay (as the coefficient of variation, ranging from 2.5 to 9.5%) were considered acceptable and allowed the quantification of phenolic compounds and their metabolites (quantified as equivalents of the respective parent molecules). The recovery ranged between 96% and 103% in plasma and between 92% and 97% in urine samples.

### 2.6. Nutrikinetic and Statistical Analysis

Statistical analyses were carried out using the program SPSS (version 23.0, SPSS, Inc., IBM Company, New York, NY, USA). Significant differences between metabolites excreted in urine after consumption of CC and CC-PP cocoa products were evaluated based on non-parametric Wilcoxon test (*p* < 0.05). To determine the absorption and elimination of epicatechin metabolites after consumption of the soluble cocoa products, metabolite nutrikinetics were studied using the pharmacokinetic functions of Microsoft Excel, calculating the maximum concentration (C_max_), area under curve (AUC) and time to reach maximum concentration (T_max_). Data are expressed as mean ± standard deviation.

## 3. Results

### 3.1. Phenolic Content of Soluble Cocoa Products 

The total polyphenolic content of both cocoa products, according to the Folin–Ciocalteau assay, was 21.70 and 25.63 µg gallic acid equivalents (GAE)/g in the conventional (CC) and flavanol-rich (CC-PP) soluble cocoa products, respectively.

In addition, the phenolic composition of both cocoa products was analyzed by HPLC-DAD. The 200 mL serving prepared from 15 and 25 g of CC and CC-PP products, respectively, provided 68.2 and 235.1 μmoles (19.80 and 68.25 mg) of flavan-3-ols, respectively (Table 1; Table S1; Figure S1). As expected, the amount of flavanols was higher in the phenol-enriched CC-PP product than in the conventional cocoa (CC), contrary to the results obtained by the Folin-Ciocalteau assay. Epicatechin was the most abundant monomer in both products (43.2% and 42.1% of the total polyphenols in CC and CC-PP, respectively) compared to catechin (24.2% and 19.4% of the total polyphenols in CC and CC-PP, respectively). Regarding dimeric procyanidins, procyanidin B2 (PB2) was present in higher concentrations than catechin, with lower amounts of procyanidin B1 (PB1) (3.0–8.5% of the total polyphenols in CC and CC-PP, respectively). 

### 3.2. LC-ESI-QToF-MS Identification of Flavanols and Metabolites in Plasma and Urine

Table 2 shows the retention time (RT), molecular formula, accurate mass of the quasimolecular ion [M-H]^−^ after negative ionization, MS^2^ fragments and location (U: urine or P: plasma) of the main compounds identified in plasma and urine samples by LC-ESI-QToF-MS. Characterization of the identified compounds was supported by commercial standards and/or previously published results. 

No un-metabolized compounds originally present in both cocoa products (CC and CC-PP) were detected in plasma and urine samples. Phase II derivatives of epicatechin were detected in biological fluids after consuming both types of soluble cocoa products. In particular, glucuronidated epicatechin was assigned to the chromatographic peak that eluted at 7.8 min, with a quasimolecular ion at *m*/*z* 465.1038 and fragment ion at *m*/*z* 289 corresponding to epicatechin, present in both plasma and urine. Based on previous studies [4,16], this peak was assigned as epicatechin-3′-glucuronide. 

Related to glucuronide derivatives, the presence of the epicatechin-methoxy-glucuronide derivative ([M-H]^−^ at *m*/*z* 479.1195 and fragment ion at *m*/*z* 303 corresponding to methoxy derivative of epicatechin) was confirmed in urine. This compound showed a higher RT (8.0 min) than that described for the glucuronidated derivative (RT at 7.8 min), consistent with the lipophilicity that the methyl group provides to the molecule. This metabolite was tentatively assigned as epicatechin-3′-methoxy-glucuronide based on the study carried out by Actis-Goretta et al. [16]. Subsequently, the chromatographic peak present in both plasma and urine at 9.8 min was assigned to epicatechin-3′-sulfate, thanks to its MS spectrum ([M-H]^−^ at *m*/*z* 369.0286 and fragment ion at *m*/*z* 289 corresponding to epicatechin). This metabolite has already been described after consumption of dark chocolate [16] and cocoa [4]. Three methoxy-sulfated isomers at 11.0, 11.7 and 12.3 min ([M-H]^−^ at *m*/*z* 383.0442 and fragment ion at *m*/*z* 303) were tentatively identified as epicatechin-methoxy-sulfate (isomer 1, isomer 2, and isomer 3), based on previous studies [4,16]. The two metabolites that eluted earlier (at 11.0 and 11.7 min) have been detected in both plasma and urine, while the third metabolite at 12.3 min was only detected in urine.

After the intake of soluble cocoa products, derivatives of phenyl-γ-valerolactone are important compounds formed as a result of the microbial metabolism of flavanols [17]. Thus, 5-(3′,4′-dihydroxyphenyl)-γ-valerolactone (DHPVL) was identified in both plasma and urine at RT 10.8 min a quasimolecular ion at *m*/*z* 207.0663 and fragment ion at *m*/*z* 163, in agreement with the pattern fragmentation already described by other authors [17,18]. In addition, phase II derivatives of DHPVL were detected. Two glucuronidated (RT at 7.4 and 8.4 min) and one sulfated (RT 12.0 min) derivatives of DHPVL were detected in both plasma and urine, except the glucuronidated metabolite at RT 7.4 min, not found in plasma. Quasimolecular ions at *m*/*z* 383.0984 and 287.0231 were compatible with glucuronide and sulfate derivatives, respectively, in addition to common fragment ions at *m*/*z* 207 and 163 corresponding to DHPVL. Finally, chromatographic peaks at 7.4, 8.4, and 12.0 min were assigned as 5-(3′-hydroxyphenyl)-γ-valerolactone-4′-glucuronide (HPVL-4′-glucuronide), 5-(4′-hydroxyphenyl)-γ-valerolactone-3′-glucuronide (HPVL-3′-glucuronide), and 5-(4′-hydroxyphenyl)-γ-valerolactone-sulfate (HPVL-sulfate), respectively, based on Actis-Goretta et al. [16], who used similar chromatographic conditions and observed that the glucuronidated and sulfated isomers of epicatechin at the C4′ position elute earlier than those at the C3′ position, contrary to what occurs with methoxy derivatives [16]. Likewise, results reported by Ottaviani et al. [8] confirmed the identity of these metabolites. 

Methoxy derivatives of DHPVL were also identified, in particular, 5-phenyl-γ-valerolactone-methoxy-glucuronide (PVL-methoxy-glucuronide) was assigned to the chromatographic peak that eluted at 8.6 min, with a quasimolecular ion at *m*/*z* 397.1140 and fragment ion at *m*/*z* 221 corresponding to 5-phenyl-γ-valerolactone-methoxy, found in plasma and urine. The chromatographic peak present in urine at 12.0 min was assigned to 5-phenyl-γ-valerolactone-methoxy-sulfate (PVL-methoxy-sulfate) thanks to its MS spectrum, ([M-H]^−^ at *m*/*z* 301.0387 and fragment ion at *m*/*z* 221). 

Based on the preferred dehydroxylation route in the C4′ of ring B described for flavanols [19], the presence of 5-(3′-hydroxyphenyl)-γ-valerolactone (HPVL) and its phase II derivatives was postulated. The search for the [M-H]^−^ ion at *m*/*z* 191.0714 yielded a peak at RT 11.6 min supported by the fragment ion at *m*/*z* 147, which allowed the identification of HPVL. Likewise, the peaks at RT 9.4 and 11.7 min showed a consistent fragmentation pattern with a glucuronidated derivative ([M-H]^−^ at *m*/*z* 367.1035 and fragment at *m*/*z* 191) and a sulfated derivative ([M-H]^−^ at *m*/*z* 271.0282 and fragment at *m*/*z* 191), respectively. These compounds have been tentatively identified as 5-(phenyl)-γ-valerolactone-3′-glucuronide (PVL-3′-glucuronide) and 5-(phenyl)-γ-valerolactone-3′-sulfate (PVL-3′-sulfate), respectively. 

Phenyl-γ-valerolactones evolve to phenylvaleric acid derivatives. Thus, 4-hydroxy-5-(3′,4′-dihydroxyphenyl)valeric acid (HDHPVA) was assigned to the chromatographic peak at RT 5.5 min detected in urine, due to its quasimolecular ion [M-H]^−^ at *m*/*z* 225.0768 and MS/MS fragmentation pattern coinciding with that described by Stoupi et al. [20], who identified this compound after in vitro fermentation of epicatechin and PB2 (fragment ion at *m*/*z* 179). Likewise, its glucuronidated derivative ([M-H]^−^ at *m*/*z* 401.1089 and fragment ion at *m*/*z* 225, corresponding to its precursor HDHPVA) and sulfated derivative ([M-H]^−^ at *m*/*z* 305.0337 and fragment ion at *m*/*z* 225) were identified, eluting at 5.1 and 7.3 min, respectively. While the sulfated metabolite was detected in both plasma and urine, the glucuronidated derivative was only detected in urine. 

Finally, derivatives of hydroxyphenylpropionic, hydroxyphenylacetic, hydroxybenzoic, and hydroxyhippuric acids were detected in plasma and urine samples (Table 2). 

### 3.3. Quantification of Plasma Metabolites and Nutrikinetic Parameters

**Figure 1:**
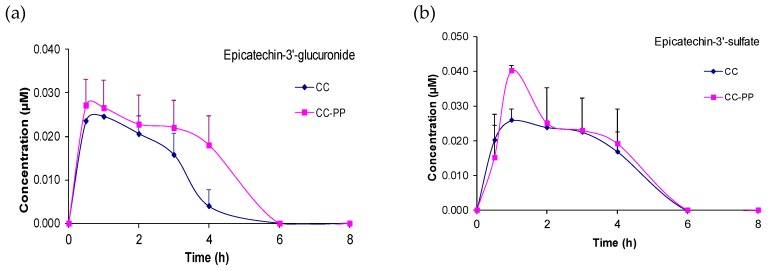

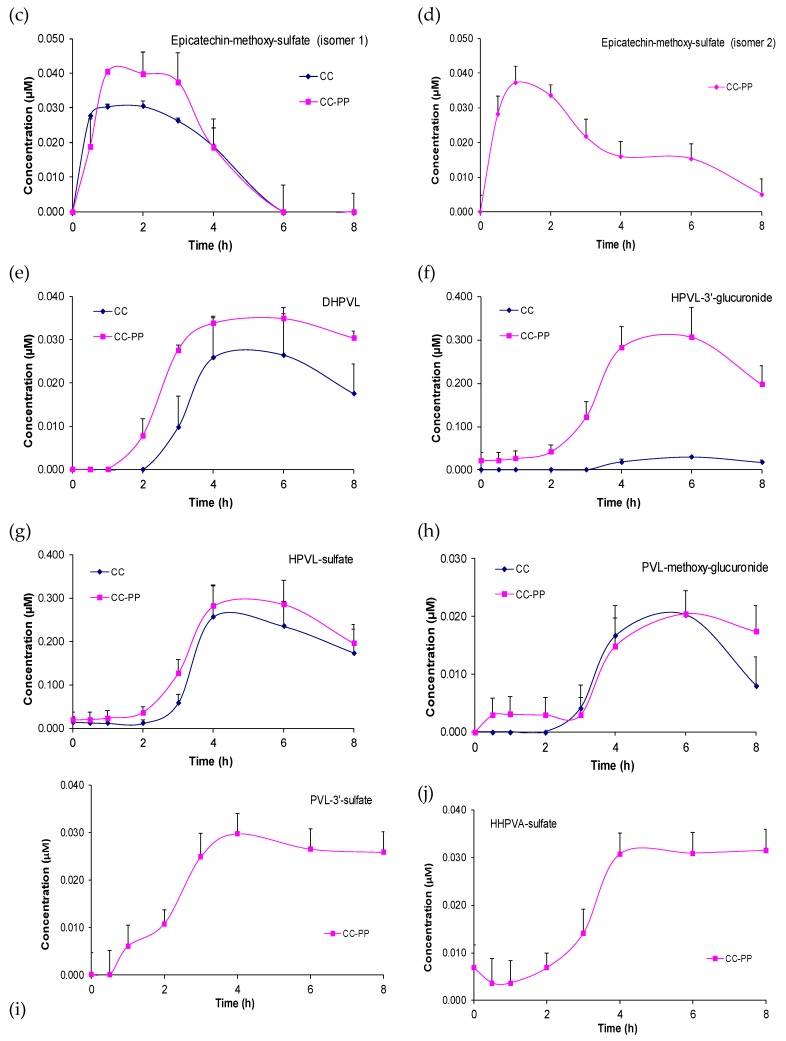
Plasma concentrations of the identified metabolites after consuming a conventional soluble cocoa product (CC) and a flavanol-rich soluble cocoa product (CC-PP) containing 19.80 and 68.25 mg of flavanols, respectively. (**a**) Epicatechin-3′-glucuronide; (**b**) epicatechin-3′-sulfate; (**c**) epicatechin-methoxy-sulfate (isomer 1); (**d**) epicatechin-methoxy-sulfate (isomer 2); (**e**) 5-(3′,4′-dihydroxyphenyl)-γ-valerolactone (DHPVL); (**f**) 5-(4′-hydroxyphenyl)-γ-valerolactone-3′-glucuronide (HPVL-3′-glucuronide); (**g**) 5-(hydroxyphenyl)-γ-valerolactone-sulfate (HPVL-sulfate); (**h**) 5-phenyl-γ-valerolactone-methoxy-glucuronide (PVL-methoxy-glucuronide); (**i**) 5-phenyl-γ-valerolactone-3′-sulfate (PVL-3′-sulfate) and (**j**) 4-hydroxy-5-(hydroxyphenyl)valeric acid-sulfate (HHPVA-sulfate). Results represent concentration (µM) as mean ± standard deviation (*n* = 13). The lower part of the error bars is not displayed for the sake of clarity.

Out of the 30 metabolites identified after consumption of the two soluble cocoa products, 8 and 10 were detected in plasma after CC and CC-PP consumption, respectively, although only 7 and 10 metabolites, respectively, showed levels above the limit of quantification. The kinetics of plasma appearance and clearance of these metabolites up to 8 h post-intake are represented in Figure 1. Nutrikinetic parameters are summarized in Table 3.

Un-metabolized flavanols were not detected in plasma after the consumption of both products (CC and CC-PP). Phase II derivatives of flavanols, epicatechin-3′-glucuronide, epicatechin-3′-sulfate and epicatechin-methoxy-sulfate (isomer 1) after CC and CC-PP intake, and epicatechin-methoxy-sulfate (isomer 2) after CC-PP intake, were detected in plasma. Concentrations of these phase II derivatives showed a rapid increase between 1 and 1.5 h after the consumption soluble cocoa products, whilst their clearance was slow, maintaining or even showing a second maxima between 3 and 6 h, with subsequent clearance at 8 h post-intake (Figure 1).

5-(3′,4′-dihydroxyphenyl)-γ-valerolactone (DHPVL) and its phase II derivatives; HPVL-3′-glucuronide, HPVL-3′-sulfate, PVL-methoxy-glucuronide, and PVL-3′-sulfate, formed the main group of metabolites detected in plasma (Figure 1). The plasmatic profile of these metabolites showed maxima concentrations between 4.9 and 6.3 h (T_max_) post-intake, except PVL-3′-sulfate after CC intake which appeared at traces level (Table 3). HPVL-3′-sulfate was the predominant metabolite after CC and CC-PP intake, showing C_max_ value of 1.150 and 1.384 μM, respectively, followed by HPVL-3′-glucuronide with C_max_ ranging from 0.106 to 1.433 μM, respectively (Table 3). 

A sulfated derivative of phenylvaleric acid, HHPVA-sulfate, was also detected in plasma but only after CC-PP intake with similar kinetic behavior than derivatives of phenyl-γ-valerolactones, with maxima concentration at 6.3 h post-intake (Figure 1). 

In general, metabolites’ C_max_ after CC consumption were significantly lower than after CC-PP intake (*p* < 0.05), consistent with the higher content of flavanols ingested with the polyphenol-rich cocoa. 

### 3.4. Quantification of Urinary Metabolites

Up to 29 and 30 metabolites were quantified in 24 h urine samples after CC and CC-PP consumption, respectively (Table 4; Table 5). Neither epicatechin nor PB2 dimer was detected in urine in any of the interventions. 

Phase II derivatives of epicatechin were preferentially excreted in the first two sampling intervals (0–4 and 4–8 h) after the ingestion of both soluble products. In the range of 0–4 h, the excretion of these metabolites was 58.3% and 62.0% (CC and CC-PP, respectively) of the total phase II derivatives of epicatechin excreted in urine; these percentages decreased to 36.0% and 34.8% in the second interval (4–8 h). Sulfated and/or methoxysulfated derivatives contributed 93% in both interventions with CC and CC-PP, showing that sulfation was the preferential route of biotransformation according to what was previously observed in the plasma. This group of metabolites represented 35.9% and 31.0% of the total urinary metabolites after CC and CC-PP intake, respectively.

DHPVL and its phase II derivatives, HPVL-4′-glucuronide, HPVL-3′-glucuronide and HPVL-sulfate, along with PVL-methoxy-glucuronide, PVL-methoxy-sulfate, PVL-3′-glucuronide and PVL-3′-sulfate conformed the most important group of metabolites quantified in urine after the ingestion of both soluble cocoa products. This group was largely excreted between 4 and 8 h post-intake and represented the 54.4% and 56.1% of the total urinary metabolites after CC and CC-PP intake, respectively. Excretion of HPVL-sulfate added up to 9.9 and 31.32 μmoles in 24 h followed by PVL-3′-sulfate (2.0 μmoles and 7.8 μmoles) after CC and CC-PP intake, respectively, evidencing that the sulfation was the preferential biotransformation pathway. 

Regarding phenylvaleric acid derivatives, HHPVA-sulfate and HHPVA-glucuronide were also excreted preferentially between 4 and 8 h post-intake, accounting for 9.7% and 12.9% of the total urinary metabolites after CC and CC-PP intake, respectively.

Finally, derivatives of hydroxyphenylpropionic, hydroxyphenylacetic, and hydrophenylbenzoic acids, along with hydroxyhippuric acid, were present in basal urine before cocoa product intake but their levels increased after CC and CC-PP consumption (0–24 h), peaking between 4 and 8 h compared to baseline values (Table 4). However, there was little difference in the total content excreted for this group of metabolites (13.1 μmoles and 15.8 μmoles after CC and CC-PP consumption, respectively) despite the large difference of polyphenol intake between both soluble cocoa products (68.2 μmoles of CC and 235 μmoles of CC-PP). For this reason, this group of metabolites was not taken into account to determine flavanols recovery. 

Attending to these results, it may be summarized that the total amount of metabolites in 24 h urine after the intake of a single serving of CC and CC-PP beverages added up to 24.1μmol and 81.3 μmoles (Table 4 and Table 5), respectively, corresponding to 35.3% and 34.6% of the 68.2 and 235.1 μmoles of polyphenols consumed, respectively.

## 4. Discussion

Relevant studies on the absorption and metabolism of flavanols in different cocoa products have been recently published [3,13,14,21]. However, the particularity of the present work lies in the realistic dose administered to the participants, following the recommendations of the cocoa product manufacturer. Furthermore, the present study compares the bioavailability of flavanols in two cocoa products: one conventional, naturally rich in cocoa (CC) and the other enriched in flavanols (CC-PP). This enabled us to check if the higher phenol doses administered with CC-PP would alter the kinetics of appearance, clearance and biotransformation. Furthermore, special attention has been paid to identify novel microbial metabolites, considering that the identification could contribute to shed light on the biotransformation pathway of flavanols in cocoa in general terms, and soluble cocoa products in particular, therefore allowing to further understand the bioactivity of these products. 

**Figure 2:**
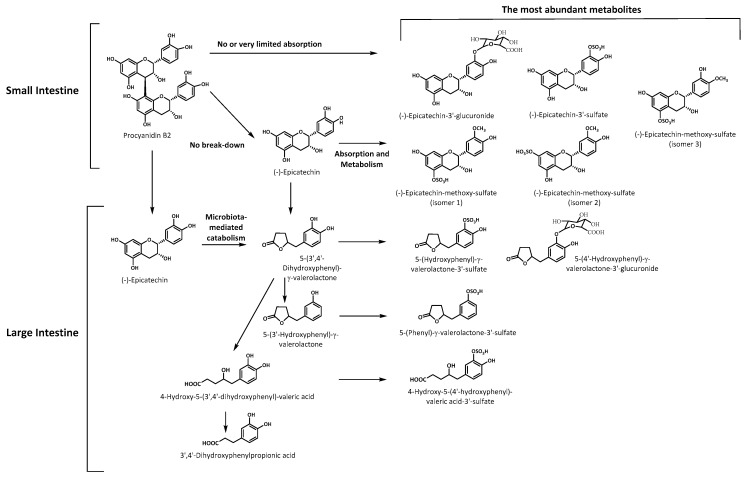
Biotransformation pathways in humans of the main flavanols contained in a conventional soluble cocoa product (CC) and a flavanol-rich soluble cocoa product (CC-PP).

The results show that flavanols present in soluble cocoa products are partially absorbed and extensively metabolized, so that most of the metabolites are produced by the intestinal microbiota. Thus, phenyl-γ-valerolactones and phenylvaleric acid derivatives, mainly as phase II conjugated metabolites, formed after absorption in the colon, were the predominant metabolites in plasma and urine, underlying the importance of the microbiota in the metabolism of flavanols (Figure 2).

Neither un-metabolized epicatechin and catechin nor procyanidins B1 and B2 were detected in the collected biological fluids in agreement with previous studies [4,11,16,22,23,24,25]. Only Schroeter et al. [26] described the presence of epicatechin and catechin in plasma after the ingestion of powdered cocoa drinks with a high flavanol content (917 mg). Flavanols followed two different pathways: a minor part of metabolites was subsequently metabolized by phase II enzymes into sulfated, glucuronidated and methoxy derivatives in the intestinal epithelium, after entering the bloodstream, whereas most flavanols reached the colon and were transformed by microbial enzymes prior to absorption and conjugation into phase II metabolites. 

Regarding the compounds identified in the systemic circulation, the present results are in agreement with those described by Actis-Goretta et al. [16], Ottaviani et al. [4], Borges et al. [21], and Montagnana et al. [11], who identified glucuronidated, sulfated and methoxy-sulfated derivatives of epicatechin in plasma. These metabolites were quantified in nM concentrations with C_max_ values from 25 to 31 nM and 37 to 42 nM after CC and CC-PP intake, respectively. As can be seen, there were no remarkable differences in the plasma concentrations of the major phase II epicatechin derivatives, in spite of the different intake of polyphenols with both cocoa products (CC, providing 19.80 mg, and CC-PP, with a total intake of 68.2 mg). In contrast, plasma concentrations as high as ~300 nM were reported by Actis-Goretta et al. [16] after the ingestion of 154 mg of flavanols in 100 g of dark chocolate, or up to 590 nM after the intake of 1,100 mg of flavanols in a soluble cocoa product [4]. Mullen et al. [25] demonstrated the interference of milk proteins in the absorption of flavanols, which might explain the lower plasmatic level of metabolites described in this study compared to Actis-Goretta et al. [16] or Ottaviani et al. [4], among others. However, it is noteworthy that these high intakes correspond to non-realistic doses that are difficult to maintain on a daily basis within a balanced diet. The time that these metabolites took to reach the maximum concentration in plasma (T_max_), ranged from 1.0 h and 1.5 h with both soluble cocoa products, pointing to the absorption in the proximal gastrointestinal tract, in agreement with other studies [4,8,24,25,27,28].

On reaching the colon, flavanols undergo microbiota-mediated conversion yielding the 5C-ring fission metabolites, 5-(hydroxypheynyl)-γ-valerolactones and 5-(hydroxyphenyl)-γ-hydroxyvaleric acids that appear in plasma preferably as phase II metabolites, being HPVL-sulfate and HPVL-3′-glucuronide the most abundant metabolites. C_max_ of the total amount of these metabolites was 424 nM and 829 nM after CC and CC-PP intake, respectively, and their T_max_ ranged from 5.0 to 6.0 h, distinctive of colon-derived products. These results are in line with those obtained by Ottaviani et al. [8], who evaluated the absorption, metabolism, distribution, and excretion of radiolabeled and stereochemically pure [2-^14^C](-)-epicatechin ([^14^C]epicatechin) in 8 male volunteers that consumed a drink containing 207 μmol (60 mg) of flavanols and reported in plasma a total concentration of 588 nM of this group of metabolites. Recently, Montagnana et al. [11] also has detected phenyl-γ-valerolactone metabolites (glucuronidated, sulfated and methoxy derivatives) in plasma 4 h after the ingestion of 50 g of 90% cocoa chocolate (7.5 gallic acid equivalents of polyphenols). 

There were substantially higher levels of metabolites in urine than plasma. The main urinary metabolites were epicatechin-methoxy-sulfate (isomer 1) and epicatechin-3′-sulfate along with epicatechin-methoxy-sulfate (isomer 3), epicatechin-3′-glucuronide and epicatechin-methoxy-sulfate (isomer 2) followed by epicatechin-3′-methoxy-glucuronide. Sulfated and/or methylsulfated derivatives of epicatechin represented 92% of this group of metabolites after consumption of both soluble cocoa products, confirming sulfation as the preferential biotransformation pathway. These results are in line with previous studies [4,8,16,25] although the proportion of metabolites was dependent on the amount of epicatechin ingested, so that a greater proportion of sulfated derivatives in all their forms (sulfated and methoxy-sulfated) was observed at lower doses of epicatechin, as in Mullen et al. [25], after the intake of 13 mg of flavanols, whereas a higher proportion of glucuronidated derivatives was observed at higher doses of epicatechin as in Ottaviani et al. [4], who administered 1100 mg of flavanols. Later Ottaviani et al. [8] detected a balanced amount of glucuronidated and sulfated derivatives of epicatechin after the intake of 60 mg of epicatechin. Phase II derivatives of epicatechin were excreted mainly in the initial 0–4 h urine collection period, followed by the interval 4–8 h and then rapidly decreased in the following intervals, in keeping with the plasma pharmacokinetic profiles (Figure 1). Phase II derivatives of epicatechin accounted for 35.9% and 31.0% of total urinary metabolites after CC and CC-PP intake, respectively, which suggests a limited bioavailability at the intestinal level. 

Regarding microbial metabolites derived from epicatechin, phase II derivatives of phenyl-γ-valerolactones and phenylvaleric acid were the most important group of metabolites quantified in urine after the ingestion of both soluble cocoa products (64.1% and 69% of the total urinary metabolites) and were largely excreted between 4–8 h post-intake. These compounds have been less described in the literature than phase II derivatives of epicatechin. Urpi-Sarda et al. [17] identified in 24 h urine mainly DHPVL and its phase II derivatives (glucuronides, sulfates, methoxy-glucuronides and methoxy-sulfates), with some being also found in plasma after the intake of 46.4 mg of flavanols in cocoa powder. Llorach et al. [29] revealed the presence of 4-hydroxy-5-(3′,4′-dihydroxyphenyl) valeric acid in 24 h urine along with some phase II derivatives of DHPVL after the consumption of a single dose of cocoa powder. Vitaglione et al. [9] also detected DHPVL in urine after the intake of different products prepared with cocoa cream. Recently, Ottaviani et al. [8] completed the characterization of phenyl-γ-valerolactones and phenylvaleric acid derivatives after consumption of 60 mg of [^14^C]epicatechin identifying sulfated and glucuronidated forms of DHPVL, HPVL and HDHPVA.

It is worth noting that excretion of HPVL-sulfate added up to 9.9 and 31.32 μmoles in 24 h after CC and CC-PP intake, respectively, evidencing that sulfation was the preferential biotransformation pathway followed by methylation and glucuronidation, in agreement with the profile described in plasma. Consequently, HPVL-sulfate as the main metabolite in both plasma and urine, could be a very sensitive biomarker of flavanol intake, considering that the excreted amount of this metabolite reached 41.1% and 38.5% of the total metabolites excreted after the consumption of CC and CC-PP, respectively. 

Finally, derivatives of hydroxyphenylpropionic, hydroxyphenylacetic, hydroxybenzoic, hydroxycinnamic, and hydroxyhippuric acids were also characterized. These compounds are not exclusively formed during the biotransformation of flavanols, since most were present in basal urine before soluble cocoa product intake (Table 4 and Table 5) and, therefore, they were not taken into account to determine flavanol recovery.

The total urinary excretion of metabolites derived from flavanols in CC and CC-PP presented a recovery of 35.3% and 34.6% of the phenols ingested, respectively, pointing to a moderate bioavailability of flavanols. It is worth noting that, although urinary recovery of metabolites, both from intestinal and colonic absorption, showed a dose-dependent increase after CC and CC-PP intake, the total amount of excreted metabolites was similar in both cases, around 35% of the ingested polyphenols, pointed out the limited bioavailability of cocoa flavanols. There is a marginal difference between the data reported in the literature regarding the recovery of flavanols. For instance, Baba et al. [27] showed an epicatechin excretion of 29.8% and 25.3% after the ingestion of chocolate and cocoa, respectively, which provided 220 mg of flavanols. Ito et al. [30] reported an excretion of 1.9% after the ingestion of 289 mg of flavanols in a soluble cocoa administered with water. Mullen et al. [25] described excretions of 18% and 10% after the ingestion of a beverage containing 13 mg of cocoa flavanols dissolved in water or milk, respectively. Afterwards, Actis-Goretta et al. [16] reported excretion values of 21.7% following the ingestion of 100 g of dark chocolate that provided 154 mg of flavanols. Recently, jejunal absorption of (−)-epicatechin in humans assessed by an intestinal perfusion technique revealed an average of ~46% (−)-epicatechin absorption based on recovery in the perfusion fluid, with high inter-individual variability among the eight volunteers participating in the study, ranging from 31% to 90% [31]. More recently, Ottaviani et al. [8] determined that the mean total recovery of radioactivity in urine from 8 volunteers in a 0–48 h period after ingestion of [^14^C]EC was 82.5 ± 4.7% of intake, with individual values ranging from 49.9% to 90.2%. In most volunteers, only a relatively small amount of radioactivity was excreted after 48 h (8; 21). It is remarkable that in all these studies there are important differences in the designs of the interventions, treatments applied to the samples before the analysis, quantification methods and even in the form of administering the flavanols (dark chocolate, milk chocolate, cocoa powder dissolved in water or milk), so that there are variables that can significantly affect the results, making comparisons difficult. Of all the factors mentioned, the matrix effect of the food has been the subject of numerous studies and, although they have generated controversial results, there are more studies that suggest an interference of milk proteins in the absorption of flavanols, as described in Mullen et al. [25], among others. High inter-individual differences in the production of metabolites have been detected in this study, as in most previous studies here cited, since different factors such as sex, age, dietary habits, and gut microbiota may significantly influence the absorption and metabolism of phenolic compounds. Among the mentioned factors, the colonic microbiota is arguably the most important factor affecting the inter-individual variability, considering that metabolites formed at colonic level constitute the predominant circulating metabolites. Nevertheless, all studies, including the present work, suggest modest recovery of flavanols in urine considering only epicatechin-derived phase II metabolites.

A limitation of this study was the reduced urinary collection time. Most of the microbial metabolites show relevant amounts in the 12–24 h interval, not returning to basal levels, and therefore it would have been interesting to extend the collection time to at least 48 h. Therefore, it is likely that the amount of urinary metabolites has been underestimated and thus a higher bioavailability of cocoa polyphenols cannot be ruled out. Another limitation was the lack of certain metabolite standards, mainly phase II derivatives, forcing to express the results as equivalents of the corresponding precursor compound. Therefore, the results here indicated did not accurately measure the concentrations of the metabolites described in the biological samples. Nevertheless, the results are in line with other studies on the bioavailability of cocoa flavanols [4,16,25]. 

In summary, polyphenols contained in two commercial, soluble cocoa products were partially absorbed and extensively metabolized. Phase II derivatives of epicatechin were identified and their pharmacokinetic profiles were compatible with epicatechin absorption at small intestine level. However, the predominant group of metabolites identified corresponded to those formed by the microbiota, hydroxyphenyl-γ-valerolatones and phenylvaleric acid, which were absorbed and metabolized into phase II derivatives. Among these metabolites, 5-(hydroxyphenyl)-γ-valerolactone 3′-sulfate showed a high amount in urine, which could be used as biomarker of intake of flavanol-rich food. In all, although flavanol bioavailability, determined by urinary recovery, showed a dose-dependent absorption after consuming both cocoa products, this was partial and limited to 35% of the ingested polyphenols, irrespective of the initial dose. 

In conclusion, the bioavailability of flavanols in soluble cocoa products is moderate, these compounds are extensively metabolized, mainly by the microbiota, and remain in the body of cocoa consumers for a long time, which favors the possible bioactivity of these compounds. 

## Figures and Tables

**Table 1:** Phenolic composition of cocoa products (conventional soluble cocoa -CC- and flavanol-rich soluble cocoa -CC-PP-) determined by high-performance liquid chromatography-diode array (HPLC-DAD).

Flavanols	CC mg/g d.m. (%)	CC-PP mg/g d.m. (%)
Epicatechin	0.57 ± 0.07 (43.2%)	1.15 ± 0.06 (42.1%)
Catechin	0.32 ± 0.03 (24.2%)	0.53 ± 0.04 (19.4%)
Procyanidin B1	0.04 ± 0.02 (3.0%)	0.23 ± 0.02 (8.5%)
Procyanidin B2	0.39 ± 0.05 (29.6%)	0.82 ± 0.06 (30.0%)
Total Flavanols	1.32 ± 0.17 (100%)	2.73 ± 0.18 (100%)

Expressed in mg per gram of dry matter (d.m. 6.5% CC and 6.7% CC-PP moisture). Values in parenthesis represent the percentage of total flavanols quantified by high-performance liquid chromatography (HPLC). Mean ± standard deviation (*n* = 3).

**Table 2:** High-performance liquid chromatography coupled to electrospray ionisation and quadrupole time-of-flight mass spectrometry (HPLC-ESI-QToF-MS) identification of flavanol metabolites detected in plasma (P) and urine (U) samples obtained after the ingestion of soluble cocoa products.

Identified Compound	RT (min)	Molecular Formula	[M-H]^−^	MS^2^ Fragment	Location
**Flavanols**					
Epicatechin-3′-glucuronide	7.8	C_21_H_22_O_12_	465.1038	289	P, U
Epicatechin-3′-methoxy-glucuronide	8.0	C_22_H_24_O_12_	479.1195	303	U
Epicatechin-3′-sulfate	9.8	C_15_H_14_O_9_S	369.0286	289	P, U
Epicatechin-methoxy-sulfate (isomer 1)	11.0	C_16_H_16_O_9_S	383.0442	303	P, U
Epicatechin-methoxy-sulfate (isomer 2)	11.7	C_16_H_16_O_9_S	383.0442	303	P, U
Epicatechin-methoxy-sulfate (isomer 3)	12.3	C_16_H_16_O_9_S	383.0442	303	U
**Phenyl-** **γ-Valerolactone (PVL) derivatives**					
5-(3′,4′-Dihydroxyphenyl)-γ-valerolactone (DHPVL)	10.8	C_11_H_12_O_4_	207.0663	163	P, U
5-(3′-Hydroxyphenyl)-γ-valerolactone-4′-glucuronide (HPVL-4′-glucuronide)	7.4	C_17_H_20_O_10_	383.0984	207;163	U
5-(4′-Hydroxyphenyl)-γ-valerolactone-3′-glucuronide (HPVL-3′-glucuronide)	8.4	C_17_H_20_O_10_	383.0984	207;163	P, U
5-(Hydroxyphenyl)-γ-valerolactone-sulfate (HPVL-sulfate)	12.0	C_11_H_12_O_7_S	287.0231	207;163	P, U
5-Phenyl-γ-valerolactone-methoxy-glucuronide (PVL-methoxy-glucuronide)	8.6	C_18_H_22_O_10_	397.1140	221	P, U
5-Phenyl-γ-valerolactone-methoxy-sulfate (PVL-methoxy-sulfate)	12.0	C_12_H_14_O_7_S	301.0387	221	U
5-(3′-Hydroxyphenyl)-γ-valerolactone (HPVL)	11.6	C_11_H_12_O_3_	191.0714	147	U
5-Phenyl-γ-valerolactone-3′-glucuronide (PVL-3′-glucuronide)	9.4	C_17_H_20_O_9_	367.1035	191	U
5-Phenyl-γ-valerolactone-3′-sulfate (PVL-3′-sulfate)	11.7	C_11_H_12_O_6_S	271.0282	191	P, U
**Phenylvaleric acid derivatives**					
4-Hydroxy-5-(3′,4′-dihydroxyphenyl)valeric acid (HDHPVA)	5.5	C_11_H_14_O_5_	225.0768	179	U
4-Hydroxy-5-(hydroxyphenyl)valeric acid-glucuronide (HHPVA-glucuronide)	5.1	C_17_H_22_O_11_	401.1089	225	U
4-Hydroxy-5-(hydroxyphenyl)valeric acid-sulfate (HHPVA-sulfate)	7.3	C_11_H_14_O_8_S	305.0337	225	P, U
***Other microbial metabolites***					
3,4-Dihydroxyphenylpropionic acid	8.5	C_9_H_10_O_4_	181.0506	137;122	P, U
3-Methoxy-4-hydroxyphenylpropionic acid	10.8	C_10_H_12_O_4_	195.0663	137	P, U
3-Hydroxyphenylpropionic acid	11.1	C_9_H_10_O_3_	165.0557	121	P, U
3,4-Dihydroxyphenylacetic acid	5.6	C_8_H_8_O_4_	167.0350	123	P, U
3-Methoxy-4-hydroxyphenylacetic acid	6.5	C_9_H_10_O_4_	181.0506	137	U
3-Hydroxyphenylacetic acid	7.4	C_8_H_8_O_3_	151.0401	107	U
Ferulic acid	12.3	C_10_H_10_O_4_	193.0506	134	P, U
*Iso*ferulic acid	15.4	C_10_H_10_O_4_	193.0506	134	U
3,4-Dihydroxybenzoic acid	3.8	C_7_H_6_0_4_	153.0193	109	P, U
4-Hydroxyhippuric acid	10.7	C_9_H_9_O_4_N	194.0459	100	P, U
3-Hydroxyhippuric acid	14.1	C_9_H_9_O_4_N	194.0459	150	P, U
Hydroxybenzoic acid	6.3	C_7_H_6_O_3_	137.0244	93	P, U

**Table 3:** Nutrikinetic parameters of metabolites detected in human plasma after consuming a conventional soluble cocoa product (CC) and a flavanol-rich soluble cocoa product (CC-PP) containing 19.80 and 68.25 mg of flavanols, respectively. Values represent mean ± standard deviation (*n* = 13).

	CC			CC-PP			*p* Value (CC vs CC-PP)		
Metabolite	C_max_ (µM)	T_max_ (h)	AUC (µM min^−1^)	C_max_ (µM)	T_max_ (h)	AUC (µM min^−1^)	C_max_	T_max_	AUC
*Intestinal absorption*									
Epicatechin-3′-glucuronide	0.025 ± 0.001	1.5 ± 0.5	0.072 ± 0.028	0.037 ± 0.001	1.4 ± 0.8	0.110 ± 0.066	<0.05	N.S.	N.S.
Epicatechin-3′-sulfate	0.026 ± 0.003	1.0 ± 0.1	0.101 ± 0.023	0.042 ± 0.004	1.2 ± 0.5	0.109 ± 0.076	N.S.	N.S.	N.S.
Epicatechin-methoxy-sulfate (isomer 1)	0.031 ± 0.003	1.3 ± 0.6	0.122 ± 0.025	0.041 ± 0.002	1.1 ± 0.6	0.173 ± 0.107	<0.05	N.S.	N.S.
Epicatechin-methoxy-sulfate (isomer 2)	N.D.			0.041 ± 0.004	1.3 ± 0.5	0.145 ± 0.107	<0.05	<0.05	<0.05
*Microbial metabolites*									
DHPVL	0.035 ± 0.008	6.0 ± 1.6	0.124 ± 0.073	0.037 ± 0.007	5.5 ± 1.4	0.192 ± 0.029	N.S.	N.S.	N.S.
HPVL-3′-glucuronide	0.031 ± 0.004	5.6 ± 0.8	0.106 ± 0.021	0.357 ± 0.200	5.0 ± 1.2	1.433 ± 0.727	<0.05	N.S.	<0.05
HPVL-sulfate	0.336 ± 0.240	5.3 ± 2.4	1.150 ± 0.742	0.332 ± 0.161	4.9 ± 1.2	1.384 ± 0.648	N.S.	N.S.	N.S.
PVL-methoxy-glucuronide	0.022 ± 0.001	5.3 ± 1.0	0.078 ± 0.032	0.027 ± 0.003	5.6 ± 1.3	0.091 ± 0.059	<0.05	N.S.	N.S.
PVL-3′-sulfate ^b^	Traces	(4–6) ^a^		0.039 ± 0.014	5.2 ± 2.2	0.161 ± 0.105	<0.05	<0.05	<0.05
HHPVA-sulfate	N.D.			0.037 ± 0.008	6.3 ± 1.8	0.167 ± 0.068	<0.05	<0.05	<0.05

AUC: area under the curve; DHPVL: 5-(3′,4′-dihydroxyphenyl)-γ-valerolactone; HPVL: 5-(3′-hydroxyphenyl)-γ-valerolactone; PVL: 5-phenyl-γ-valerolactone; HDHPVA: 4-hydroxy-5-(3′,4′-dihydroxyphenyl)valeric acid; N.D.: Not detected; N.S.: Non-significant differences; *p* values were assessed using the general linear model of variance for repeated measures. ^a^ Range where the metabolite showed the highest value. ^b^ No pharmacokinetic parameters of these metabolites were determined because they were present at trace levels.

**Table 4:** Metabolites excreted in urine (from 0 to 24 h) by healthy volunteers after consumption of the conventional soluble cocoa product (CC) containing 19.80 mg of flavanols.

	Metabolite	Basal (µmol)	0–4 h (µmol)	4–8 h (µmol)	8–12 h (µmol)	12–24 h (µmol)	Total (µmol)
**Intestinal Absorption**	Epicatechin-3′-glucuronide	N.D.	0.355 ± 0.071	0.267 ± 0.059	<L.Q.	<L.Q.	0.622 ± 0.130 *
	Epicatechin-3′-methoxy-glucuronide	N.D.	<L.Q.	<L.Q.	N.D.	N.D.	<L.Q. *
	Epicatechin-3′-sulfate	N.D.	1.323 ± 0.236	1.018 ± 0.178	0.076 ± 0.025	<L.Q.	2.417 ± 0.439 *
	Epicatechin-methoxy-sulfate (isomer 1)	N.D.	2.717 ± 0.457	1.300 ± 0.197	0.154 ± 0.031	0.257 ± 0.114	4.428 ± 0.800 *
	Epicatechin-methoxy-sulfate (isomer 2)	N.D.	0.181 ± 0.032	0.247 ± 0.030	<L.Q.	<L.Q.	0.428 ± 0.062 *
	Epicatechin-methoxy-sulfate (isomer 3)	N.D.	0.473 ± 0.089	0.285 ± 0.036	<L.Q.	N.D.	0.758 ± 0.125 *
	Total—intestinal absorption	N.D.	5.049 ± 0.885	3.117 ± 0.500	0.230 ± 0.056	0.257 ± 0.114	8.653 ± 1.556 *
**Colonic Absorption**	DHPVL	<L.Q.	<L.Q.	0.146 ± 0.032	<L.Q.	<L.Q.	0.146 ± 0.032 *
	HPVL	N.D.	N.D.	<L.Q.	<L.Q.	<L.Q.	<L.Q. *
	HDHPVA	N.D.	N.D.	<L.Q.	<L.Q.	<L.Q.	<L.Q.
	HPVL-4′-glucuronide	<L.Q.	<L.Q.	0.111 ± 0.049	<L.Q.	<L.Q.	0.111 ± 0.049 *
	HPVL-3′-glucuronide	<L.Q.	<L.Q.	0.497 ± 0.204	0.103 ± 0.028	<L.Q.	0.600 ± 0.232 *
	HPVL-sulfate	0.143 ± 0.064	0.456 ± 0.193	5.638 ± 1.922	1.945 ± 0.647	1.744 ± 0.374	9.926 ± 3.200 *
	PVL-methoxy-glucuronide	<L.Q.	<L.Q.	0.111 ± 0.039	<L.Q.	<L.Q.	0.111 ± 0.039 *
	PVL-methoxy-sulfate	<L.Q.	N.D.	0.085 ± 0.031	<L.Q.	<L.Q.	0.085 ± 0.031 *
	PVL-3′glucuronide	N.D.	<L.Q.	0.233 ± 0.155	0.126 ± 0.078	0.079 ± 0.022	0.438 ± 0.255
	PVL-3′-sulfate	<L.Q.	<L.Q.	0.860 ± 0.385	0.456 ± 0.284	0.375 ± 0.133	1.691 ± 0.802 *
	HHPVA-glucuronide	N.D.	N.D.	N.D.	N.D.	N.D.	N.D. *
	HHPVA-sulfate	0.089 ± 0.055	0.076 ± 0.055	1.112 ± 0.397	0.565 ± 0.171	0.503 ± 0.152	2.345 ± 0.830 *
	Total—colonic absorption	0.232 ± 0.119	0.532 ± 0.248	8.793 ± 3.214	3.195 ± 1.208	2.701 ± 0.681	15.453 ± 5.470 *
**Other microbial metabolites**	3,4-Dihydroxyphenylpropionic acid	0.117 ± 0.024	0.217 ± 0.051	0.243 ± 0.039	0.074 ± 0.017	0.215 ± 0.016	0.866 ± 0.148
	3-Methoxy-4-hydroxyphenylpropionic acid	0.104 ± 0.027	0.144 ± 0.044	<L.Q.	<L.Q.	<L.Q.	0.248 ± 0.071
	Hydroxyphenylpropionic acid	0.118 ± 0.023	0.226 ± 0.046	0.259 ± 0.043	0.077 ± 0.018	0.203 ± 0.025	0.883 ± 0.154
	3,4-Dihydroxyphenylacetic acid	0.094 ± 0.032	0.648 ± 0.505	0.556 ± 0.379	0.094 ± 0.033	0.133 ± 0.020	1.525 ± 0.974
	3-Methoxy-4-hydroxyphenylacetic acid	0.103 ± 0.014	0.099 ± 0.054	0.093 ± 0.028	<L.Q.	0.103 ± 0.022	0.398 ± 0.118
	Hydroxyphenylacetic acid	0.369 ± 0.077	0.408 ± 0.084	0.605 ± 0.092	0.225 ± 0.052	0.600 ± 0.100	2.208 ± 0.328
	Ferulic acid	<L.Q.	<L.Q.	0.094 ± 0.009	0.099 ± 0.059	N.D.	0.193 ± 0.068
	*Iso*ferulic acid	0.170 ± 0.137	0.086 ± 0.028	0.096 ± 0.040	0.088 ± 0.069	0.082 ± 0.016	0.522 ± 0.290
	Protocatechuic acid	0.161 ± 0.071	0.227 ± 0.142	0.373 ± 0.237	0.094 ± 0.047	0.104 ± 0.044	0.959 ± 0.217 *
	Hydroxyhippuric acid	0.821 ± 0.251	0.864 ± 0.154	1.171 ± 0.387	0.242 ± 0.067	0.995 ± 0.316	4.093 ± 1.175
	Hydroxyhippuric acid	0.075 ± 0.031	0.085 ± 0.043	0.082 ± 0.055	0.075 ± 0.031	0.077 ± 0.046	0.394 ± 0.206
	Hidroxybenzoic acid	0.188 ± 0.041	0.145 ± 0.027	0.200 ± 0.051	0.070 ± 0.018	0.195 ± 0.025	0.798 ± 0.162
	Total other microbial metabolites	2.320 ± 0.728	3.149 ± 1.178	3.772 ± 1.360	1.138 ± 0.411	2.707 ± 0.630	13.086 ± 3.911
	INTESTINAL + COLONIC METABOLITES	0.232 ± 0.119	5.581 ± 1.133	11.910 ± 3.714	3.425 ± 1.264	2.958 ± 0.795	24.106 ± 7.026

HDHPVA: 4-hydroxy-5-(3′,4′-dihydroxyphenyl)valeric acid; DHPVL: 5-(3′,4′-dihydroxyphenyl)-γ-valerolactone; HPVL: 5-(3′-hydroxyphenyl)-γ-valerolactone; PVL: 5-phenyl-γ-valerolactone. * Significant differences respect to CC-PP intervention was observed based on non-parametric Wilcoxon test at *p* < 0.05. Values represent mean ± standard deviation (*n* = 13). N.D.: not detected; <L.Q. lower than quantification limit.

**Table 5:** Metabolites excreted in urine (from 0 to 24 h) by healthy volunteers after consumption of the flavanol-rich soluble cocoa product (CC-PP) containing 68.25 mg of flavanols.

	Metabolite	Basal (µmol)	0–4 h (µmol)	4–8 h (µmol)	8–12 h (µmol)	12–24 h (µmol)	Total (µmol)
**Intestinal Absorption**	Epicatechin-3′-glucuronide	N.D.	0.958 ± 0.272	0.695 ± 0.190	<L.Q.	<L.Q.	1.653 ± 0.462 *
	Epicatechin-3′-methoxy-glucuronide	N.D.	0.121 ± 0.036	<L.Q.	<L.Q.	N.D.	0.121 ± 0.036 *
	Epicatechin-3′-sulfate	N.D.	3.627 ± 0.797	3.572 ± 0.662	0.158 ± 0.038	<L.Q.	7.357 ± 1.523 *
	Epicatechin-methoxy-sulfate (isomer 1)	N.D.	8.642 ± 1.434	3.277 ± 0.644	0.330 ± 0.095	0.232 ± 0.070	12.481 ± 2.243 *
	Epicatechin-methoxy-sulfate (isomer 2)	N.D.	0.566 ± 0.152	0.557 ± 0.120	0.077 ± 0.021	<L.Q.	1.200 ± 0.293 *
	Epicatechin-methoxy-sulfate (isomer 3)	N.D.	1.702 ± 0.243	0.652 ± 0.148	<L.Q.	<L.Q.	2.354 ± 0.402 *
	Total—intestinal absorption	N.D.	15.616 ± 2.934	8.753 ±1.764	0.565 ± 0.154	0.232 ± 0.070	25.166 ± 4.959 *
**Colonic Absorption**	DHPVL	<L.Q.	<L.Q.	0.628 ± 0.087	0.174 ± 0.029	0.282 ± 0.082	1.084 ± 0.199 *
	HPVL	N.D.	<L.Q.	0.104 ± 0.041	<L.Q.	<L.Q.	0.104 ± 0.041 *
	HDHPVA	N.D.	<L.Q.	<L.Q.	<L.Q.	<L.Q.	<L.Q.
	HPVL-4′-glucuronide	<L.Q.	<L.Q.	0.330 ± 0.111	0.550 ± 0.015	0.081 ± 0.025	0.961 ± 0.151 *
	HPVL-3′-glucuronide	<L.Q.	<L.Q.	1.802 ± 0.567	0.274 ± 0.060	0.282 ± 0.130	2.358 ± 0.766 *
	HPVL-sulfate	0.332 ± 0.164	1.965 ± 0.636	15.979 ± 2.963	5.386 ± 0.934	7.656 ± 2.125	31.318 ± 6.822 *
	PVL-methoxy-glucuronide	<L.Q.	<L.Q.	0.340 ± 0.074	0.084 ± 0.014	0.131 ± 0.046	0.555 ± 0.134 *
	PVL-methoxy-sulfate	N.D.	<L.Q.	0.177 ± 0.036	<L.Q.	<L.Q.	0.177 ± 0.047 *
	PVL-3′glucuronide	<L.Q.	0.108 ± 0.045	0.521 ± 0.191	0.179 ± 0.063	0.508 ± 0.349	1.316 ± 0.648
	PVL-3′-sulfate	<L.Q.	0.651 ± 0.389	4.188 ± 1.535	1.223 ± 0.373	1.690 ± 0.572	7.752 ± 2.868 *
	HHPVA-glucuronide	N.D.	<L.Q.	0.714 ± 0.088	0.087 ± 0.019	0.251 ± 0.075	1.052 ± 0.182 *
	HHPVA-sulfate	0.089 ± 0.032	0.584 ± 0.223	5.018 ± 1.090	1.798 ± 0.376	1.987 ± 0.566	9.476 ± 2.287 *
	Total—colonic absorption	0.421 ± 0.196	3.308 ± 1.293	29.801 ± 6.783	9.755 ± 1.883	12.868 ± 3.970	56.153 ± 14.145 *
**Other microbial metabolites**	3,4-Dihydroxyphenylpropionic acid	0.213 ± 0.031	0.256 ± 0.027	0.238 ± 0.028	0.109 ± 0.016	0.301 ± 0.029	1.117 ± 0.131
	3-Methoxy-4-hydroxyphenylpropionic acid	0.077 ± 0.032	0.175 ± 0.054	<L.Q.	<L.Q.	0.199 ± 0.052	0.461 ± 0.138
	Hydroxyphenylpropionic acid	0.074 ± 0.013	0.350 ± 0.038	0.356 ± 0.076	0.140 ± 0.041	0.284 ± 0.075	1.204 ± 0.243
	3,4-Dihydroxyphenylacetic acid	0.109 ± 0.015	0.751 ± 0.057	0.556 ± 0.096	0.198 ± 0.037	0.539 ± 0.196	2.153 ± 0.401
	3-Methoxy-4-hydroxyphenylacetic acid	0.198 ± 0.071	0.188 ± 0.026	0.124 ± 0.050	<L.Q.	0.138 ± 0.016	0.648 ± 0.178
	Hydroxyphenylacetic acid	0.585 ± 0.070	0.631 ± 0.140	0.678 ± 0.231	0.434 ± 0.070	0.534 ± 0.143	2.862 ± 0.654
	Ferulic acid	<L.Q.	0.137 ± 0.092	0.150 ± 0.094	0.151 ± 0.094	0.312 ± 0.290	0.750 ± 0.570
	*Iso*ferulic acid	<L.Q.	0.164 ± 0.044	0.160 ± 0.066	0.152 ± 0.094	0.210 ± 0.152	0.686 ± 0.356
	Protocatechuic acid	0.088 ± 0.014	0.105 ± 0.016	0.113 ± 0.020	0.095 ± 0.040	0.127 ± 0.015	0.528 ± 0.105 *
	Hydroxyhippuric acid	0.871 ± 0.264	0.789 ± 0.100	0.579 ± 0.096	0.328 ± 0.063	0.987 ± 0.193	3.554 ± 0.716
	Hydroxyhippuric acid	0.086 ± 0.013	0.090 ± 0.009	0.082 ± 0.009	0.075 ± 0.063	0.129 ± 0.017	0.462 ± 0.111
	Hidroxybenzoic acid	0.212 ± 0.037	0.396 ± 0.060	0.298 ± 0.036	0.172 ± 0.063	0.302 ± 0.169	1.380 ± 0.365
	Total other microbial metabolites	2.513 ± 0.560	4.032 ± 0.663	3.334 ± 0.802	1.854 ± 0.581	4.062 ± 1.347	15.805 ± 3.968
	INTESTINAL + COLONIC METABOLITES	0.421 ± 0.196	18.924 ± 4.227	38.554 ± 8.547	10.320 ± 2.037	13.100 ± 4.040	81.319 ± 19.104

HDHPVA: 4-hydroxy-5-(3′,4′-dihydroxyphenyl)valeric acid; DHPVL: 5-(3′,4′-dihydroxyphenyl)-γ-valerolactone; HPVL: 5-(3′-hydroxyphenyl)-γ-valerolactone; PVL: 5-phenyl-γ-valerolactone. * Significant differences respect to CC-PP intervention was observed based on non-parametric Wilcoxon test at *p* < 0.05. Values represent mean ± standard deviation (*n* = 13). N.D.: not detected; <L.Q. lower than quantification limit.

## Supplementary Materials

The following are available online at https://www.mdpi.com/2072-6643/11/7/1441/s1, Figure S1: Representative high-performance liquid chromatography-diode array (HPLC-DAD) chromatograms of phenolic compounds of a conventional soluble cocoa (CC) (upper panel, **a**) and a flavanol-rich soluble cocoa (CC-PP) (lower panel, **b**) registered at 280 nm; Table S1: Retention times and λmax of the compounds identified by high-performance liquid chromatography-diode array (HPLC-DAD) and the masses of each of the flavonoid identified determined by LC-ESI-QToF-MS.
