# Supplementary material for: Flavanol Bioavailability in Two Cocoa Products with Different Phenolic Content. A Comparative Study in Humans
Source: Nutrients. 2019 Jun 26;11(7):1441. doi: 10.3390/nu11071441 (PMC6683251; doi:10.3390/nu11071441)
Supplement: Supplementary file 1 [file nutrients-11-01441-s001.pdf]

Figure S1. Representative high-performance liquid chromatography-diode array (HPLC-DAD) chromatograms of phenolic compounds of a conventional soluble cocoa (CC) (upper panel, **a**) and a flavanol-rich soluble cocoa (CC-PP) (lower panel, **b**) registered at 280 nm.

(a)

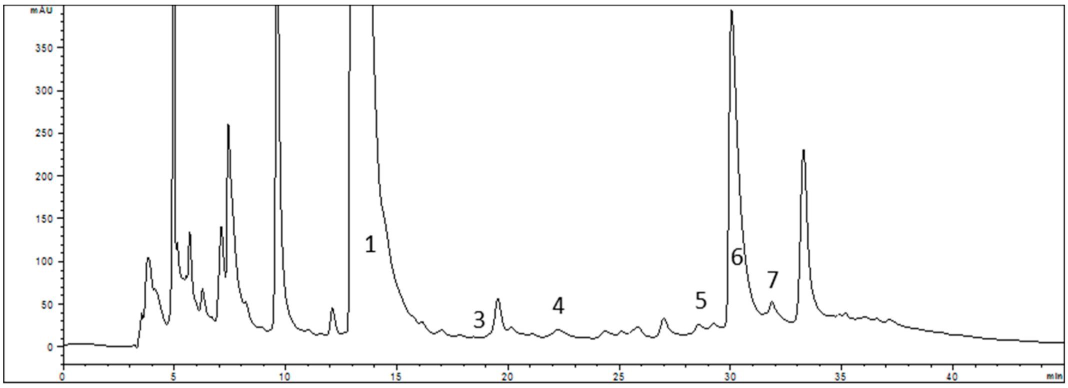

(b)

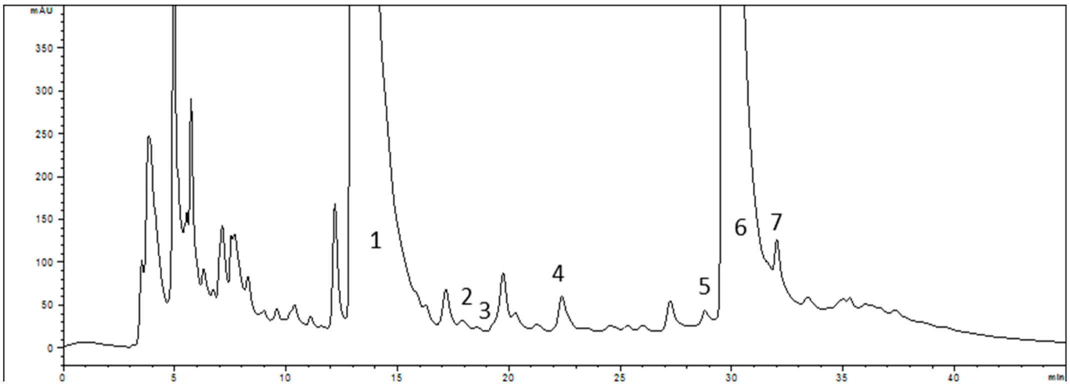

Table S1. Retention times and λmax of the compounds identified by high-performance liquid chromatography-diode array (HPLC-DAD) and the masses of each of the flavonoid identified determined by high-performance liquid chromatography coupled to electrospray ionisation and quadrupole time-of-flight mass spectrometry (HPLC-ESI-QToF-MS).

Peak	Name	RT (min)	λ max	$[M-H]^-$	Fragments ions
1	Theobromine	13.5	272	-	-
2	Theophylline	18.0	272	-	-
3	Procyanidin B1	18.8	280	577	289
4	Catechin	22.4	280	289	245
5	Procyanidin B2	28.9	280	577	289
6	Caffeine	30.4	272	-	-
7	Epicatechin	32.0	280	289	245
